# Editorial: Natural Product Epigenetic Modulators and Inhibitors

**DOI:** 10.3389/fphar.2021.651395

**Published:** 2021-03-04

**Authors:** Fidele Ntie-Kang, Berin Karaman Mayack, Sergio Valente, Cecilia Battistelli

**Affiliations:** ^1^ Molecular Simulations Laboratory, Department of Chemistry, University of Buea, Buea, Cameroon; ^2^ Institute of Pharmacy, Martin-Luther University of Halle-Wittenberg, Halle (Saale), Germany; ^3^ Institute of Botany, Technical University Dresden, Dresden, Germany; ^4^ Department of Pharmaceutical Chemistry, Istanbul University Istanbul, Istanbul, Turkey; ^5^ Department of Drug Chemistry and Technologies, Faculty of Pharmacy and Medicine, Sapienza University of Rome, Rome, Italy; ^6^ Department of Molecular Medicine, Sapienza University of Rome, Rome, Italy

**Keywords:** chromatin, drugs, epigenetics, inihibition, methylation

Epigenetics in drug discovery have recently drawn a lot of attention and there are increasing number of applications as reflected in the growing number publications and citations on this subject. This is because epigenetic dysfunction is widely implicated in several human diseases, including cancer, neurodegenerative and parasitic diseases. Epigenetic broadly involves covalent changes on the building blocks of chromatins called nucleosomes. The reactions involved include methylation, acetylation, phosphorylation, ubiquitination and reverse reactions of those. Natural products (NPs) are regaining attention as sources of lead compounds that can be used as a starting point of drug or epigenetic probe discovery. Besides, NPs have documented large structural diversity with unique and complex scaffolds with promising activity and selectivity profile with epigenetic targets. This special collection of articles is dedicated to the research topic “Natural Products Epigenetic Modulators and Inhibitors.” This is a focused research area that covers NPs and NP mimics and derivatives, which play important modulatory or inhibitory roles in the epigenome.

NPs and natural remedies have a potential role to play in the treatment of several diseases, including age-related and neurodegenerative disorders. A detailed summary of the health benefits of NPs capable of inhibiting or modulating the zinc-independent histone deacetylases (HDACs), sirtuins, varying from anti-aging to cardiovascular disease, weight loss, etc. have been summarized in a recent review article (Karaman Mayack et al., 2020). Among the sirtuins, sirtuin 1 (sirt1) is the most studied member, and its expression has been associated with the increase in insulin sensitivity. Besides, sirt1 is known to be implicated in tumorigenic and anticancer processes, as well as in the regulation of some essential metabolic pathways by controlling p53 deacetylation and modulation of autophagy. Targeting Sirt1 could affect caloric restriction and extend human lifespan. NPs (including polyphenols contained in products like fruits, vegetables, and plants) have recently been found to upregulate sirt1 activity (Iside et al.). Besides, epigenetic mechanisms (particularly histone deacetylase-inhibition) have been recently reported to be involved in epilepsy and chronic pain development (De Caro et al.), as well in several other disease-associated epigenetic mechanisms, including cancer (Cassandri et al.; Karaman Mayack et al., 2020). For this reason, several NPs have been repurposed for the treatment of different diseases and cancer itself (Montalvo-Casimiroet al. 2020; Moreira-Silvaet al. 2020).

Several recent chemoinformatics studies have been conducted with the goal of exploring the chemical space and target space of NPsthat play epigenetic roles in nature (Sessions et al., 2020), including inhibitors of DNA methyltransferases (DNMTs) (Saldívar-González et al.). The chemical and activity landscape of naturally occurring epigenetic modulation and inhibition has often motivated lead compound discovery for these targets to virtually screen NP databases (Naveja and Medina-Franco 2015; Naveja et al., 2018; Akone et al.). A survey of the structure-multitarget activity relationships and diversity (>7,000 compounds, including NPs), which had been previously tested against ∼50 epigenetic targets showed that HDACs and other epigenetic targets are clustered in the chemical space, whereas the chemical space of inhibitors of the various DNMTs included in the study did not overlap. This observation indicated the selectivity of DNMTs toward their inhibitors (Naveja and Medina-Franco 2018). In addition, inhibitors of HDACs are known to reverse latency in the human immunodeficiency virus (HIV), facilitating antiviral activity to target and kill the virus in the “chock and kill” process (Andersen et al., 2018; Divsalar et al.).

In the interesting review by Akone and collaborators (Akone et al.), the authors focus on NPs that inhibit DNA methyltransferases (DNMTs) and histone deacetylases (HDACs). Notably they emphasize that these natural drugs can be considered as promising candidates in the treatment of cancer. While DNA methylation and histone modifications represent epigenetic signatures regulating gene expression (Allis and Jenuwein, 2016) in physiological events (development, differentiation, proliferation), hypomethylation and hypermethylation of DNA have been observed in cancer cells (Qi and Xiong, 2018). For this reason, the identification of highly specific and not toxic compounds represents a main topic in the field. The most known drugs targeting DNMTs are aza-cytidine and decitabine. Despite their usefulness as epigenetic modulators, these compounds are highly toxic and poorly stable (Gnyszka et al., 2013). More recently, some natural compounds have been reported as efficient against DNMTs activity; specifically, in the aforementioned review, the authors extensively describe the activity of (–)-epigallocatechin-3-gallate, contained in green tea, polyphenol curcumin, the flavonoid quercetin, kazinol Q, resveratrol (3, 4′, 5-trihydroxystilbene), the quinone Nanaomycin A, the isoflavone genistein, the isothiocyanate sulforaphane, the pentacyclic terpenoid Boswellic acid, the *Z*-ligustilide, the germacrane sesquiterpene lactone parthenolide and the ubiquinone derivative Antroquinonol D. Moreover, they approach the analysis of HDACs inhibitors, due to their important role in cancer progression treatment (Lee et al., 2017). As previously described, also in the case of HDACs inhibitors, natural compounds represent a starting point for the development of structures that could be highly selective for the different HDAC isoforms and potentially active in cancer. Trichostatin A was the first natural HDAC inhibitor described but its lack of selectivity limits its use (Khan et al., 2008; Shen and Kozikowski, 2016). More recently, different compounds have been designed and synthetized and some of them show good efficacy in cancer cells and in different patho-physiological states (Rossi et al., 2018). Specifically, the distinct HDAC inhibitors differ between zinc-binding inhibitors (linear HDAC inhibitors, cyclic tetrapeptides, cyclic depsipeptides) and non-zinc-binding inhibitors (e.g., Ursolic acid, Epicocconigrones A and B, Curcumin, *n*-Butyric acid and Aceroside VIII). Overall, in this manuscript (Akone et al.) the authors highlight the importance of NPs that can represent a basis for the development of specific and efficient new-generation compounds.

In line with this paper, Fiorentino and co-workers (Fiorentino et al.) analyze the role of a distinct class of acetyltransferases, the lysine acetyltransferases (KATs), normally involved in cell signaling, metabolism, gene regulation, and apoptosis, that transfer acetyl groups on target proteins. It is well-known that alteration of KATs enzymatic activity correlates with different pathological states (inflammation, neurological diseases and cancer). For these reasons the conceptualization, design and synthesis of KAT inhibitors (KATi) could represent a powerful strategy to counteract these conditions. In this field of investigation, NPs have been identified as active compounds counteracting KATs. Specifically, the authors extensively report that NPs, despite their low selectivity, represent suitable scaffolds for the development of new derivatives potentially more specific and with a high grade of activity. With this intent, Fiorentino et al., make an overview starting from garcinol and isogarcinol that represented the starting point for the development of new molecules. Then they discuss about epigallocatechin-3-gallate, a non-selective KATi, active against p300, CBP, PCAF and Tip60 (Choi et al., 2009), delphinidin andcurcumin, despite it cannot be considered a selective KATi. The dissertation is enriched with the focus on other NPs differentially active against KATs. Specifically, the authors analyze the potentiality of different quinones, the 6-pentadecylsalicylic acid (anacardic acid) and its derivatives, that show different activity especially on CBP and p300. Starting from this evidence, the authors discuss also the activity and effectiveness of alkaloids, possessing inhibitory activity against p300 and PCAF as well as the role of prostaglandins and peptide metabolites. In summary, different NPs represent a good starting point for the development of new drugs in the field of KAT inhibitors with low off-target effects and high efficacy.
